# Photoelectrochemical Behavior and Computational Insights for Pristine and Doped NdFeO_3_ Thin-Film Photocathodes

**DOI:** 10.1021/acsami.0c21792

**Published:** 2021-03-17

**Authors:** Javier Quiñonero, Francisco J. Pastor, José M. Orts, Roberto Gómez

**Affiliations:** ^†^Departament de Química Física, ^‡^Institut Universitari d’Electroquímica, Universitat d’Alacant, Apartat 99, E-03080 Alicante, Spain

**Keywords:** NdFeO_3_, photoelectrochemistry, doping, photocathode, DFT

## Abstract

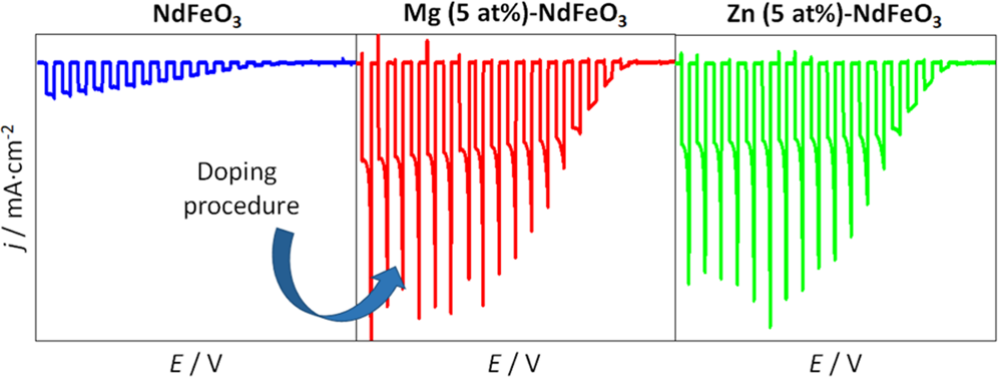

Among the different strategies that are being developed to solve the current energy challenge, harvesting energy directly from sunlight through a tandem photoelectrochemical cell (water splitting) is most attractive. Its implementation requires the development of stable and efficient photocathodes, NdFeO_3_ being a suitable candidate among ternary oxides. In this study, transparent NdFeO_3_ thin-film photocathodes have been successfully prepared by a citric acid-based sol–gel procedure, followed by thermal treatment in air at 640 °C. These electrodes show photocurrents for both the hydrogen evolution and oxygen reduction reactions. Doping with Mg^2+^ and Zn^2+^ has been observed to significantly enhance the photoelectrocatalytic performance of NdFeO_3_ toward oxygen reduction. Magnesium is slightly more efficient as a dopant than Zn, leading to a multiplication of the photocurrent by a factor of 4–5 for a doping level of 5 at % (with respect to iron atoms). This same trend is observed for hydrogen evolution. The beneficial effect of doping is primarily attributed to an increase in the density and a change in the nature of the majority charge carriers. DFT calculations help to rationalize the behavior of NdFeO_3_ by pointing to the importance of nanostructuring and doping. All in all, NdFeO_3_ has the potential to be used as a photocathode in photoelectrochemical applications, although efforts should be directed to limit surface recombination.

## 1 Introduction

In the current context of growing global energy demand and depletion of fossil fuels, photoelectrochemical (PEC) water electrolysis driven by sunlight on semiconductor electrodes could be a suitable avenue for the sustainable generation of H_2_.^1−5^ In fact, hydrogen is identified as a technically viable and carbon-free energy vector for applications ranging from small-scale power supply to large-scale energy storage and transportation.^6−8^

Since the seminal demonstration of photoelectrochemical hydrogen production with a cell comprising TiO_2_ and Pt electrodes by Fujishima and Honda,^9^ revolutionary advances made in the field of PEC water-splitting systems have led to H_2_ production with solar-to-hydrogen efficiencies higher than 10%^10^ but using relatively complex device architectures and/or electrodes made of rare and/or unstable materials. However, the development of viable, simple photoelectrochemical tandem cells is hindered by difficulties in finding efficient, stable, and inexpensive photocathodes.^10,11^

In this regard, several binary and ternary metal oxides, such as CuO (*E*_g_ = 1.0 eV),^12,13^ Cu_2_O (*E*_g_ = 2.1 eV),^14,15^ CaFe_2_O_4_ (*E*_g_ = 1.9 eV),^16,17^ CuBi_2_O_4_ (*E*_g_ = 1.8 eV),^18,19^ and CuFeO_2_ (*E*_g_ = 1.5 eV),^20,21^ can behave as p-type semiconductors. However, none of them meets the criteria to be considered as practical photocathodes in water-splitting devices (chemical photostability in aqueous environments, efficient light absorption, and low cost). These facts, along with the low values for charge carrier mobility and charge carrier lifetime that usually characterize oxide photocathodes, make critical further research on this topic.

Among ternary oxides, perovskite-type (ABO_3_) oxides^22−26^ may constitute suitable candidates as photocathodes because they generally exhibit remarkable stability under illumination in aqueous solutions and some of them present a p-type semiconducting behavior. LaFeO_3_, the flagship of this family of materials in PEC applications, has been widely identified as a promising electrode material according to several studies on its photoelectrochemical characterization.^27−31^ In fact, its band-gap value is reported to be around 2.1 eV, which makes possible the absorption of a substantial portion of the visible solar spectrum.^32^ To intensify its p-type character, doping with metals with a predominant +2 oxidation state and ionic radii similar to that of Fe^3+^ (such as Mg^2+^ and Zn^2+^) has been revealed as an effective strategy with applications in both photoelectrochemistry^33^ and photocatalysis.^34^

On the other hand, in the field of water splitting on semiconductor electrodes, theoretical and computational studies based on DFT calculations have gained importance.^35^ In fact, they provide valuable information regarding key aspects, such as band-gap values, band structures, charge transport and transfer, as well as the kinetics of oxygen and hydrogen evolution reactions at the electrode/electrolyte interface.^36^ Thus, DFT has become a useful tool that contributes substantially to the understanding of the photoactive materials’ response, and it has oriented the optimization of the PEC performance through proper modeling and simulation strategies.^37−39^

In the present study, based on the previous work done with the LaFeO_3_ perovskite, a characterization of NdFeO_3_ photocathodes is presented. As far as we know, this is the first photoelectrochemical study on this material, even though Nd is as abundant as La in the Earth’s crust. Specifically, NdFeO_3_ electrodes prepared on transparent conductive substrates by a straightforward sol–gel procedure are shown to exhibit cathodic photocurrents for H_2_O and O_2_ reduction over a wide range of potentials. Their magnitude, particularly for oxygen reduction, is significantly intensified by the partial substitution of Fe in the perovskite structure by either Mg or Zn impurities. DFT calculations have also been performed to gain insights into the physics underlying the p-type photoelectrochemical behavior of both pristine and doped NdFeO_3_ electrodes.

## 2 Experimental Section

### 2.1 Preparation of Pristine NdFeO_3_ Photocathodes

Pristine NdFeO_3_ thin-film photocathodes were prepared on fluorine-doped tin oxide conducting glass (TEC 15, 12–15 Ω/□, Pilkington) following a previously reported procedure based on a citric acid-based sol–gel route.^33^ The NdFeO_3_ precursor solution was prepared by dissolving Nd(NO_3_)_3_·6H_2_O (Aldrich Chemistry, 99%) 0.3 M and Fe(NO_3_)_2_·9H_2_O (Panreac, 98%) 0.3 M in water (Millipore, Elix 3). This solution was magnetically stirred for 1 h and then the appropriate amount of citric acid (Merck, 99.5%) was added to reach a concentration of 0.6 M. The resulting solution was stirred for 20 h. Next, 30 μL·mL^–1^ acetylacetone (Fluka Analytical, 99.5%) and 30 μL·mL^–1^ Triton X-100 (laboratory grade, Sigma-Aldrich) was added. An FTO glass plate (area to be covered: 1 cm^2^) was cleaned by sonication (Selecta Ultrasonics) for 15 min in acetone (VWR Chemicals, 99%) and ethanol (VWR Chemicals, 96%). It was then spin-coated (Chemat Technology, KW-4A) at 1500 rpm for 20 s with 40 μL of the precursor solution. Finally, the electrodes were annealed in air at 500 °C for 1 h using a programmable furnace (Conatec, 7800), with a heating rate of 5 °C·min^–1^. This process was repeated four times to obtain the optimized film thickness (four layers, see Figure S1) and a final thermal treatment at 640 °C was applied for 2 h to obtain the crystalline perovskite films.

### 2.2 Preparation of Doped NdFeO_3_ Photocathodes

Mg (*x* at %)- and Zn (*x* at %)-doped (*x* = 1; 2.5; 5 and 10) NdFeO_3_ thin-film photocathodes were obtained by following the same procedure described above but substituting the corresponding amount of the Fe precursor salt in the NdFeO_3_ precursor solution with the dopant salt in the desired concentration (in at % with respect to the Fe content). In this case, Mg(NO_3_)_2_·6H_2_O (VWR Chemicals, 99.4%) and Zn(NO_3_)_2_·6H_2_O (Fluka, 99.0%) were employed as Mg and Zn precursors, respectively.

### 2.3 (Photo)electrochemical Measurements

(Photo)electrochemical measurements were conducted at room temperature in a home-made Pyrex glass cell with a fused silica window with a computer-controlled potentiostat-galvanostat (Autolab, PGSTAT30). A Pt wire and a Ag/AgCl/KCl(3 M) electrode were used as counter and reference electrodes, respectively, and an N_2_- or O_2_-purged 0.1 M NaOH (Panreac, 98.0%) solution was used as the working electrolyte. Unless otherwise stated, all potentials are referred to the Ag/AgCl/KCl(3 M) electrode. The light source was an ozone-free 1000 W Xe(Hg) lamp (Newport Instruments, 66921) equipped with a water filter to minimize the infrared contribution. Unless otherwise stated, the photoelectrochemical measurements were carried out by irradiating the photocathodes through the electrolyte/electrode interface (EE illumination). The photoresponse under this type of illumination is higher than that obtained under substrate–electrode (SE) illumination (see Figure S2). The light intensity reaching the electrode was measured by means of a photodiode power meter (Thorlabs, PM100D), and it was adjusted to 100 mW cm^–2^.

Details regarding equipment and measurement conditions for X-ray diffraction (XRD), scanning electron microscopy (SEM), X-ray photoelectron spectroscopy (XPS), and UV–vis spectroscopy are given in the SI.

### 2.4 Computational Methods

**Figure 1:**
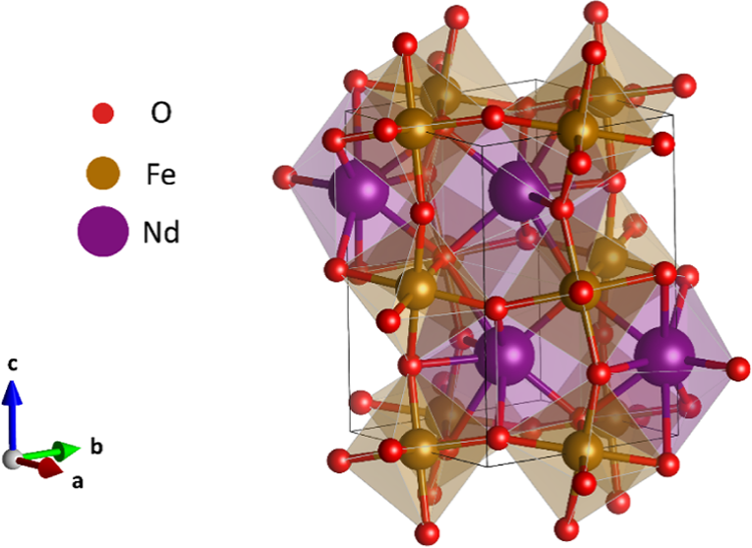
Standard primitive orthorhombic NdFeO_3_ cell used for DFT calculations.

Density functional theory (DFT) calculations for both pristine and doped NdFeO_3_ have been performed with the Vienna ab initio simulation package (VASP),^40^ under the projector augmented wave (PAW)^41,42^ method with a plane-wave energy cutoff of 520 eV for all of the calculations. The number of valence electrons used was 11 for Nd (5s^2^5p^6^5d^1^6s^2^), eight for Fe (3d^7^4s^1^), six for O (2s^2^2p^4^), two for Mg (2s^2^2p^0^), and 12 for Zn (3d^10^4p^2^). The model geometry was a standard primitive orthorhombic cell (SG number 62) for pristine NdFeO_3_ calculations, with a total of 20 atoms, as seen in Figure 1. For doped NdFeO_3_ calculations, we have used a 2x2x1 supercell in which an Fe atom was replaced by either a Mg or a Zn atom (6.7 at %). The Perdew–Burke–Ernzerhof (PBE)^43^ generalized gradient approximation (GGA) functional has been used together with the single-parameter approach enunciated by Dudarev et al.^44^ to include the effect of the electronic correlation of the highly localized d-electrons in these transition metal oxides, with a *U* value of 4.0 eV for the Fe d-states.^45^ In addition, the hybrid Heyd–Scuseria–Ernzerhof (HSE06)^46,47^ functional (which is known to provide good estimates of the electronic structure) has been used to check and complement the results obtained with PBE + *U* (see details in the SI). The pristine NdFeO_3_ calculations have been made with a gamma-centered Monkhorst-Pack-type 5 × 5 × 3 *k*-mesh for geometry optimization and with an 11 × 11 × 7 *k*-mesh for the density of state (DOS) calculations. For the former, the electronic convergence criterion was set at 10^–6^ eV, while forces on atoms were set to be below 0.01 eV Å^–1^ for geometry convergence. The calculations for doped NdFeO_3_ have been carried out with a 3 × 2 × 4 *k*-mesh for geometry optimization and with a 5 × 8 × 5 *k*-mesh for DOS calculations, using the same convergence criteria as for pristine NdFeO_3_ calculations. The Brillouin zone has been integrated with the tetrahedron method with Bloch corrections. All calculations have been made with spin-polarization, adopting a G-type antiferromagnetic arrangement for the Fe atoms.^48^ No spin–orbit coupling has been considered. Band-structure calculations have been performed for both pristine and doped NdFeO_3_ perovskites. From the calculated band structure for pristine NdFeO_3_, the effective masses of charge carriers have been obtained by means of the Boltztrap2 code.^49^

## 3 Results and Discussion

### 3.1 Physical and Chemical Characterization of Pristine and Doped NdFeO_3_ Photocathodes

**Figure 2:**
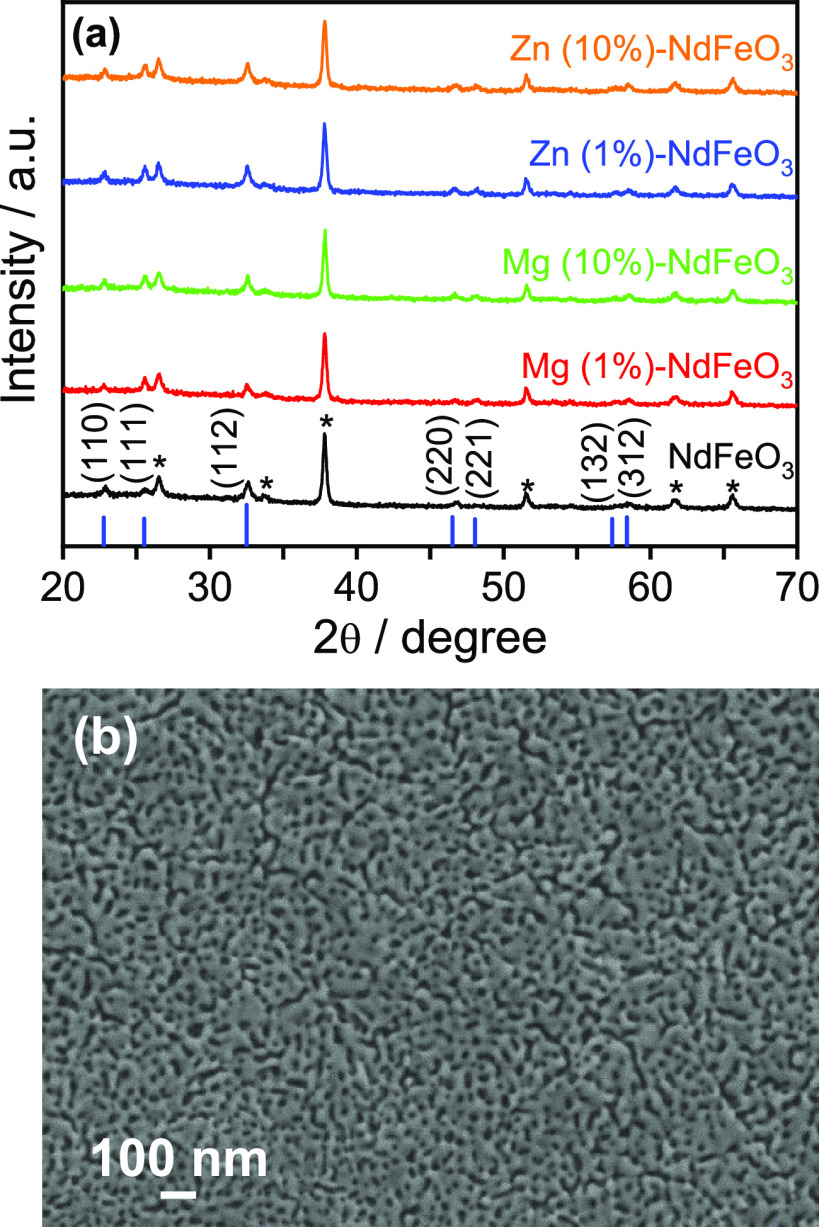
(a) XRD patterns for NdFeO_3_, Mg (1 at %)-NdFeO_3_, Mg (10 at %)-NdFeO_3_, Zn (1 at %)-NdFeO_3_, and Zn (10 at %)-NdFeO_3_ films on FTO substrates (peaks marked with asterisks correspond to the SnO_2_ substrate). (b) FESEM image corresponding to the top view of an NdFeO_3_ film on FTO.

Figure 2a shows the XRD patterns for pristine and Mg (*x* at %)- and Zn (*x* at %)-doped (*x* = 1 and 10) NdFeO_3_ thin films deposited on an FTO conductive glass substrate. Apart from FTO, NdFeO_3_ is the only crystalline phase detected in the film (ICDD card no. 74-1473). The XRD analysis confirms that the yellowish-brown NdFeO_3_ films are crystalline, presenting an orthorhombic phase with lattice parameters *a* = 5.453 Å, *b* = 5.584 Å, and *c* = 7.768 Å and α = β = γ = 90°. There are no important changes in the XRD patterns upon magnesium and zinc doping, although there is a small shift of less than 0.1° in the (112) reflection position toward smaller 2θ values upon doping, particularly in the case of Mg. Thus, the orthorhombic phase is maintained with some distortion after the incorporation of either Mg or Zn. This fact is consistent with the relatively small difference in the ionic diameters for Fe^3+^ (78.5 pm), Mg^2+^ (86 pm), and Zn^2+^ (88 pm)^50^ and also with reports that confirm the prevalence of the substitution of the Fe atoms in the perovskite structure by Mg or Zn, instead of the formation of new phases, at least for dopant concentrations up to 30%.^34,51−53^

Figure 2b shows a representative FESEM image of a pristine NdFeO_3_ sample. As observed, it consists of a nanocrystalline deposit with a certain degree of porosity (intercrystalline pores around 30 nm in size). A cross-sectional FESEM image (Figure S3) reveals that the films have an average thickness of around 300 nm, constituted by individual nanocrystals with an approximate diameter of 60 nm. Representative FESEM images of Mg (*x* at %)- and Zn (*x* at %)-doped (*x* = 5) NdFeO_3_ deposits are shown in Figure S4. From these images, it can be deduced that an optimum doping level (in terms of photoelectrochemical performance, see below) does not induce any significant alteration in the topographic and surface morphological characteristics of the synthesized thin films. In fact, only an apparently larger grain size can be discerned for doped samples.

**Figure 3:**
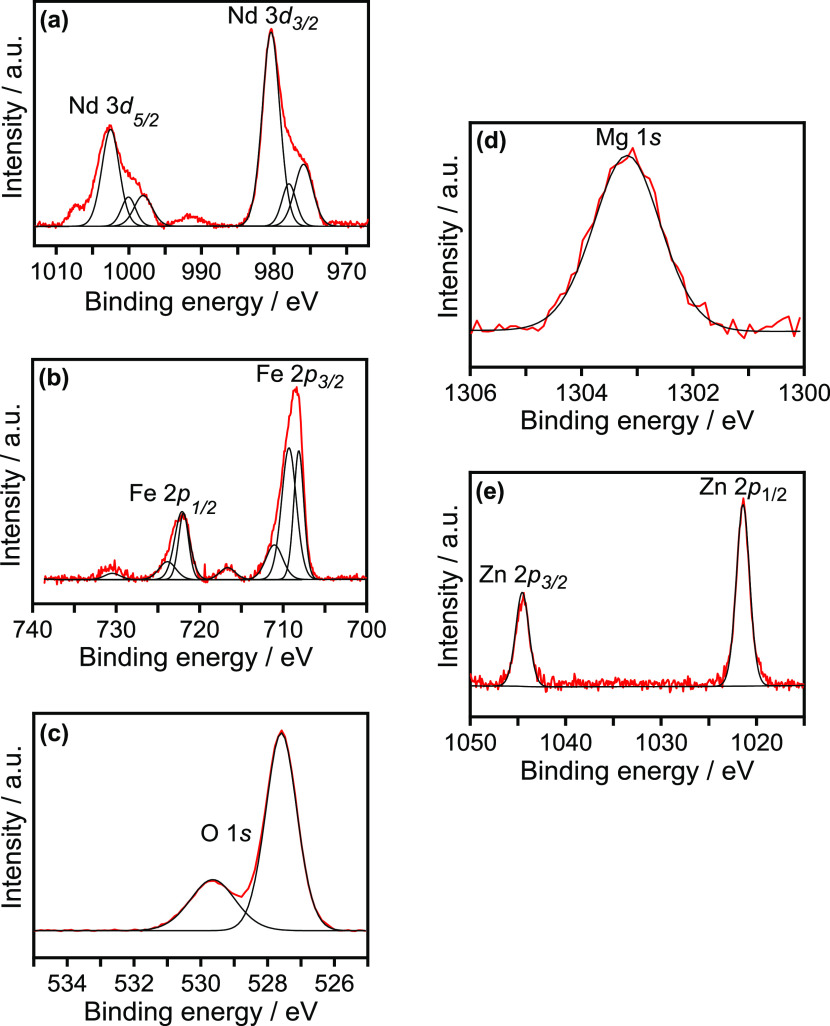
(a) Nd 3d, (b) Fe 2p, and (c) O 1s XPS spectra (red line) and corresponding deconvolutions (black lines) for an NdFeO_3_ film on FTO. (d) Mg 1s and (e) Zn 2p XPS spectra (red lines) and corresponding deconvolutions (black lines) for Mg (5 at %)-NdFeO_3_ and Zn (5 at %)-NdFeO_3_ films on FTO.

Figure 3a–c shows XPS spectra for the as-prepared pristine NdFeO_3_ sample. The Nd 3d XPS spectrum (Figure 3a) shows two asymmetric peaks at binding energies (BEs) of 1002.5 and 980.4 eV, attributed to the 3d_5/2_ and 3d_3/2_ transitions, respectively, with shoulders (interpreted as two satellite peaks) separated from the corresponding main peak by around 4.5 eV. This complex, characteristic spin–orbit splitting confirms the presence of Nd^3+^ in the sample.^54,55^ The Fe 2p XPS spectrum (Figure 3b) exhibits two main peaks at BEs of 708.5 and 722.0 eV corresponding to the Fe 2p_3/2_ and Fe 2p_1/2_ transitions, respectively.^33^ Although the satellite peak at 716.6 eV is a further indication of the presence of Fe^3+^ in the films,^33,56^ the existence of minor amounts of Fe^2+^ cannot be ruled out due to the BE values observed for Fe^3+^, which are slightly lower than those usually reported.^33^ The O 1s XPS spectrum (Figure 3c) can be deconvoluted into two different components: the first one, at a BE of 528.6 eV, is ascribed to oxide species in the perovskite lattice,^57^ while the peak at 529.6 eV is assigned to surface hydroxyl groups, which suggests a hydroxylated surface for the NdFeO_3_ deposit.^57,58^ In this regard, it is interesting to point out that certain lanthanide oxides (such as La_2_O_3_) can adsorb water rapidly with a subsequent conversion of surface oxide ions to yield a hydroxylated surface.^57,58^

The XPS analysis of the doped samples confirms the presence of either Mg or Zn at the surface. In the Mg 1s spectrum (Figure 3d) for the corresponding doped films, there appears a signal at 1303.2 eV, while peaks are present at 1044.6 and 1021.4 eV in the Zn 2p spectrum (Figure 3e) corresponding to the Zn 2p_3/2_ and 2p_1/2_ transitions, respectively.^33^ The XPS spectra for Nd 3d, Fe 2p, and O 1s for Mg (5 at %)- and Zn (5 at %)-doped NdFeO_3_ deposits (Figures S5 and S6) are virtually identical to those obtained for the pristine samples. However, it is interesting to note that the surface ratios of Mg/Fe and Zn/Fe in the Mg (5 at %)- and Zn (5 at %)-NdFeO_3_ deposits are between five and six times those expected in the bulk (Table S1). Such evident segregation could be correlated with the increase in the OH surface concentration in the doped electrodes, as the oxygenated Mg and Zn species are more hygroscopic than the Fe ones.^33^

**Figure 4:**
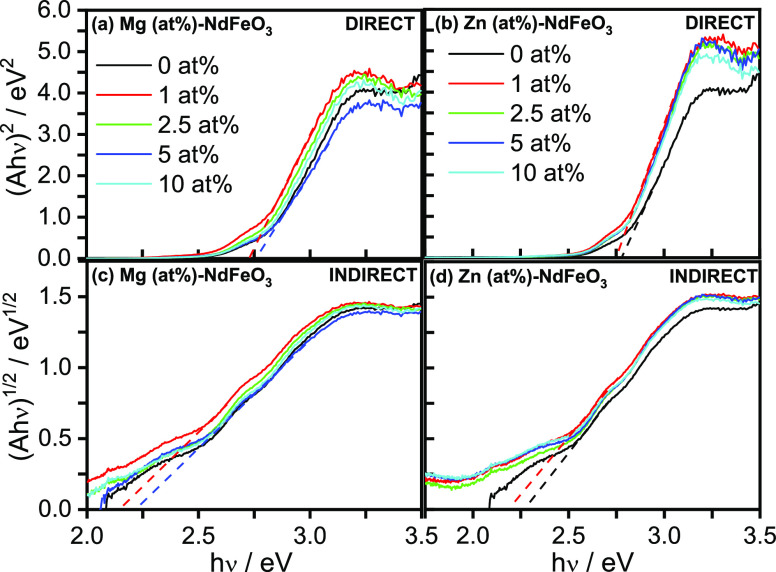
Direct and indirect Tauc plots for (a, c) Mg (*x* at %)-NdFeO_3_ and (b, d) Zn (*x* at %)-NdFeO_3_ electrodes (absorbance values have been corrected for the contribution of the FTO conducting glass substrate).

Figure 4 shows direct and indirect Tauc plots for pristine and Mg- and Zn-doped NdFeO_3_ thin films, obtained from the corresponding UV–visible absorbance spectra (Figure S7), characterized by an absorption edge at around 600 nm. The absorbance curves are similar for all of the samples, irrespective of the doping level. Direct and indirect optical band-gap values of 2.8 and 2.2 eV, respectively, can be calculated from Figure 4. Doping does not significantly alter either direct or indirect band-gap values, which are in all cases within a 0.1 eV range.

### 3.2 (Photo)electrochemical Characterization of Pristine and Doped NdFeO_3_ Photocathodes

**Figure 5:**
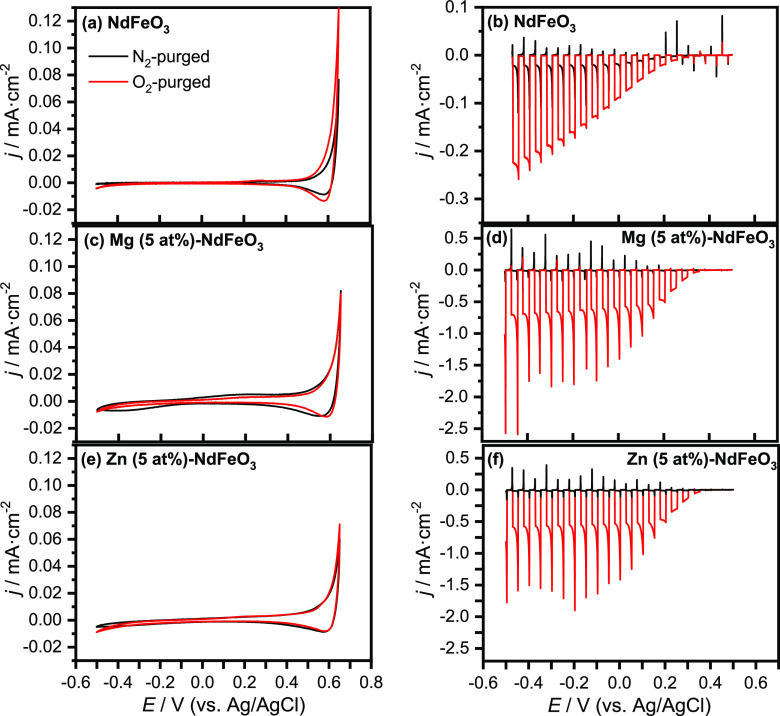
Cyclic voltammograms in the dark in N_2_- and O_2_-purged 0.1 M NaOH for (a) NdFeO_3_, (b) Mg (5 at %)-NdFeO_3_, and (c) Zn (5 at %)-NdFeO_3_ electrodes (scan rate: 20 mV·s^–1^). Linear scan voltammograms under transient illumination in N_2_- and O_2_-purged 0.1 M NaOH for (d) NdFeO_3_, (e) Mg (5 at %)-NdFeO_3_, and (f) Zn (5 at %)-NdFeO_3_ electrodes (scan rate: 5 mV·s^–1^).

Cyclic voltammograms in the dark for pristine NdFeO_3_ photocathodes in N_2_- and O_2_-purged 0.1 M NaOH (Figure 5a) reveal the existence of small capacitive currents over a wide potential window, while the region at potentials above 0.2 V can be assimilated to the accumulation region for a p-type electrode.^59,60^ No significant changes in the corresponding cyclic voltammograms in the dark are observed in the presence of O_2_. The small but stable cathodic photocurrents observed in linear scan voltammograms under transient illumination in an N_2_-purged working electrolyte (Figure 5b) are indicative of H_2_ evolution from water reduction by the photogenerated electrons, further confirming the p-type character of the as-prepared semiconductor electrodes. As observed, the onset potential of the photocurrent is located at around 0.2 V. Since the onset of the photocurrent is close to that of the capacitive currents in the dark, such an accumulation region can be related to the filling/emptying of valence band states.^59,60^ Remarkably, the shape of the transients points to electron-trapping at the electrode surface with the appearance of cathodic spikes upon illumination and anodic ones when the light is switched off, which is indicative of an electron–hole recombination. In fact, electron-trapping at the surface is one of the most important factors limiting the performance of perovskite-based photocathodes.^61^ On the contrary, the presence of an electron scavenger such as O_2_ in the working electrolyte induces a drastic increase in photocurrent (by around 10 times) and a diminution of the spikes in the illumination transients, which indicates significant mitigation of surface-electron-trapping. Importantly, photocurrents in both N_2_- and O_2_-purged electrolytes are remarkably stable over time as shown in transients recorded for 10 min of chopped illumination at 0.1 V for pristine and Mg (5 at %)- and Zn (5 at %)-doped NdFeO_3_ electrodes (Figure S8 in the SI).

To study the doping effects on the (photo)electrochemical properties of NdFeO_3_ electrodes, cyclic voltammograms in the dark and linear scan voltammograms under transient illumination for Mg- and Zn-doped NdFeO_3_ photocathodes were recorded. As observed in Figure 5c,e (and Figures S9a and S10a), doping with Mg and Zn does not induce important changes in the voltammetric response of the NdFeO_3_ electrodes in the dark in either N_2_- or O_2_-purged working electrolytes. Only in the presence of O_2_, the capacitive region at potentials above 0.2 V seems to present a more defined shape. As the charge in the accumulation region slightly increases upon doping, so does the electrochemically active surface area. Such a slight increase is not expected to impact significantly the photoelectrode response.

Linear scan voltammograms under transient illumination in a N_2_-purged electrolyte exhibit the typical response of the pristine NdFeO_3_ photocathode, with no significant spike intensity or photocurrent onset location. However, there is a significant increase in the photocurrent magnitude as clearly shown in the photocurrent transients of Figure S8. Higher enhancements in the photocurrent values are observed upon Mg and Zn doping (Figure 5d,f, respectively) in the presence of an electron scavenger such as O_2_ (see also Figure S8). This enhancement in photocurrent is clearly dependent on the NdFeO_3_ doping level (Figures S9b and S10b). As observed in Figure S11, the stationary photocurrent at 0.1 V increases with the dopant concentration until an optimum value is reached at *x* = 5 at % for both Mg and Zn. The fact that the photocurrent values and their dependence on the dopant concentration are similar for both Mg and Zn indicates that the role of these cations as dopants in the perovskite structure should be equivalent.

**Figure 6:**
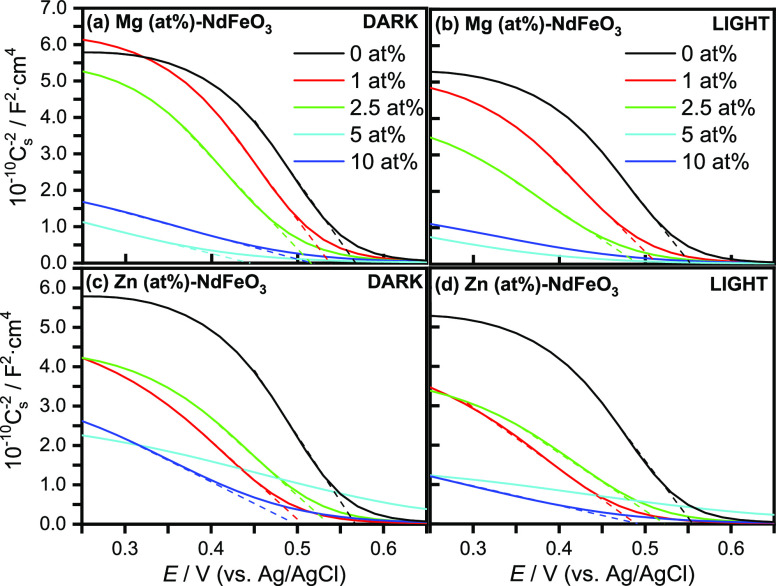
Mott–Schottky plots in the dark and under illumination for (a, b) Mg (*x* at %)-NdFeO_3_ and (c, d) Zn (*x* at %)-NdFeO_3_ electrodes in N_2_-purged 0.1 M NaOH.

Mott–Schottky analysis in the dark and under illumination (Figure 6) has been performed to further confirm the p-type character of the as-prepared pristine and doped NdFeO_3_ photocathodes and to gain some insights into the mechanism for photocurrent enhancement by Mg and Zn. In fact, the 1/*C*^2^ vs *E* plots show, although only in a limited range of potential, the expected tendency for a p-type semiconductor under band edge pinning conditions (linear with a negative slope). From the intercepts with the abscissa axis, the flat band potential (*E*_fb_) for the pristine NdFeO_3_ photocathode can be estimated to be around 0.57 V both in the dark and under illumination. This indicates that the bands are not unpinned under illumination, which suggests that electron accumulation at the surface is not substantial. As the slope of the straight line in the Mott–Schottky plot is inversely proportional to the density of the majority charge carriers (*N*_A_), the plots show how the charge carrier density increases under illumination. Considering a dielectric constant (ε) of 75,^62^ the calculated *N*_A_ for the pristine NdFeO_3_ electrode in the dark and under illumination is 6.3 × 10^18^ and 7.4 × 10^18^ cm^–3^, respectively.

For the Mg- and Zn-doped samples, no significant changes of the *E*_fb_ value are observed. In fact, only a minor shift toward less positive potentials (not desirable in the case of photocathodes) can be deduced. Thus, the potentials of the conduction and valence band edges should be similar for both pristine and doped electrodes according to the small variations in *E*_fb_ and band-gap values. Therefore, the enhancement of the photoresponse cannot be ascribed to an increase in the reducing power of the photogenerated electrons and/or in the fraction of visible light absorbed by the electrode. The progressive decrease in the slope values as the Mg and Zn dopant densities increase (Table S2) indicates that such an enhancement can be related to an increase in the density of majority charge carriers (*N*_A_) in the material. This effect is especially notable for the highest densities of Mg and Zn (although they do not exhibit the best photoelectrochemical performance).

### 3.3 Computational Results

**Figure 7:**
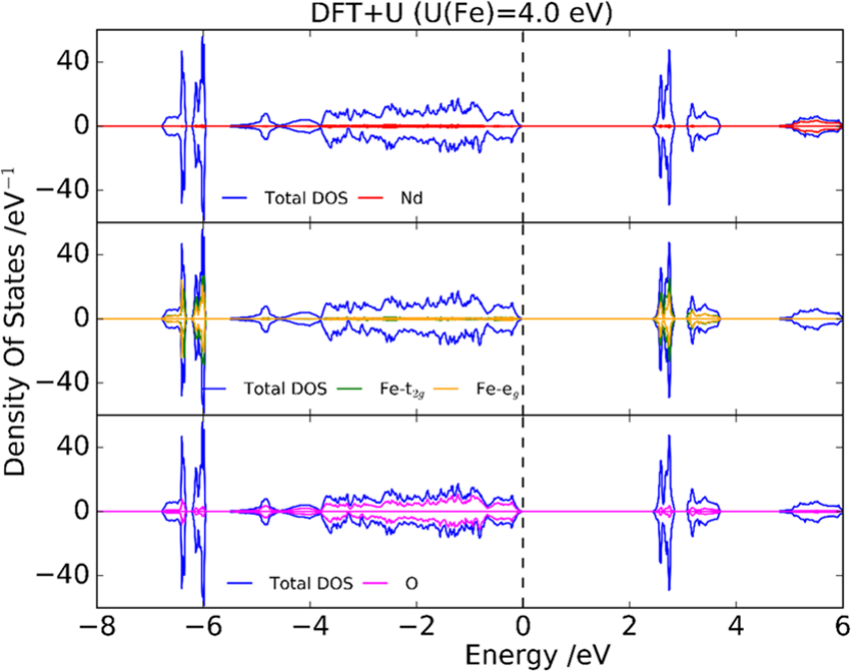
Total and partial density of states for NdFeO_3_ at PBE + *U* (*U* (Fe) = 4.0 eV) (the dashed line corresponds to the Fermi level, which has been shifted to zero).

Figure 7 shows the total and partial density of states in the case of NdFeO_3_ for PBE + *U* (*U* (Fe) = 4.0 eV). The distribution of the total DOS is very similar to that obtained with the HSE06 (α = 15%) functional (see Figure S12), defining direct band-gap values of 2.4 and 3.0 eV with PBE+U and HSE06, respectively. Considering that the experimental direct band gap is around 2.8 eV, a reasonable agreement is obtained with these two theory levels. On the contrary, there is no evidence for the experimental indirect band-gap value, suggesting that it originates from transitions implying defect states rather than from interband transitions.

There is no significant contribution of Nd to the total DOS close to the band edges. In this respect, there is experimental evidence that the main contributions to the valence and conduction bands are from Fe and O states.^33,63^ According to PBE + *U* (and HSE06), the occupied states of Fe are very localized and very deep in energy, although there is also a small contribution to the total DOS close to the Fermi level. In the case of O, the computational results show that the O 2p states are mainly distributed in the range from −5 eV up to the Fermi level. It can also be seen that there is an important hybridization between Fe 3d and the O 2p orbitals both above and below the Fermi level.

**Figure 8:**
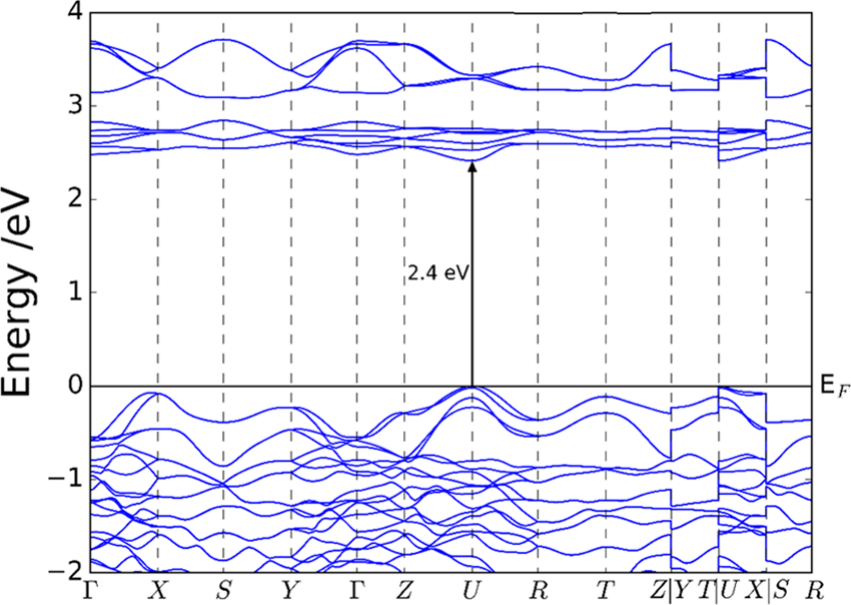
Band structure of NdFeO_3_ calculated with the DFT+*U* model.

Figure 8 shows the band structure of NdFeO_3_ calculated at the PBE+*U* level. The conduction band edge is rather flat, which indicates that the charge carriers (in this case, electrons) possess very low mobility. In this respect, Table 1 shows the effective mass values for electrons and holes along the crystal axis directions, obtained with Boltztrap2 from PBE+U band-structure calculations (see Table S3 for results obtained from the HSE06 band structure). It is worth noting that for both charge carriers, the effective masses show an anisotropic behavior. This means that the orientation of the crystals on the substrate is thus relevant as it should influence the photoelectrochemical response. In materials with anisotropic conductivity, it is highly advantageous to have nanostructures preferentially oriented in the most convenient crystal direction, such as nanosheets or nanorods. However, in this case, the preferential direction is the same for both charge carriers. The fact that, irrespective of the direction, the effective mass of CB electrons is twice that of VB holes can explain in part that (i) the semiconductor exhibits a p-type behavior and (ii) the photocurrents obtained for EE illumination are larger than those for SE illumination (Figure S2). This can be rationalized if we consider that the electrodes possess a space charge region. For EE illumination, photogenerated holes need to travel through the bulk until they reach the electric contact, while for SE illumination, the electron is the charge carrier that should cross the thin film before reaching the electrode–electrolyte interface.

**Table 1:** Calculated Band Gap and Effective Masses for Carriers in NdFeO_3_ from the Band Structure (PBE + *U*)

calculation	band gap/eV	charge carrier	m*_a_/m_e_	m*_b_/m_e_	m*_c_/m_e_
PBE + *U*	2.4	electron	11.0	2.8	8.7
		hole	1.8	1.4	3.0

DFT calculations provide evidence that the upper edge of the valence band consists mainly of O 2p states, with a minor contribution of the Fe 3d e_g_ states. According to this, photogenerated holes in NdFeO_3_ can be ascribed to the O^–^ and Fe^4+^ species.^33^ In fact, the electric p-type nature of certain AFeO_3_ perovskites is thought to arise from the presence of small amounts of Fe^4+^ in their structure, compensated by the corresponding level of excess oxygen and resulting in an AFeO_3+*x*_ stoichiometry.^64^ In the case of LaFeO_3_ obtained by a sol–gel method using the citrate route (similar to that used here for the preparation of NdFeO_3_), the fraction of Fe^4+^ over total Fe was estimated to be between 2.4 and 3.2%.^51^ On the other hand, the lower edge of the conduction band consists mainly of Fe 3d t_2g_ and e_g_ states with a minor contribution of O 2p states, contrary to what happens with the upper edge of the valence band.

Thus, the increase in the NdFeO_3_ acceptor level density and photoactivity upon Mg and Zn doping can be explained considering a subsequent increase in the concentration of Fe^4+^ species: the substitution of trivalent iron cations by divalent doping elements (Mg^2+^ and Fe^2+^, in this case) may induce, through a charge compensation mechanism, either the corresponding oxidation of Fe^3+^ to Fe^4+^ or a partial loss of excess oxygen (which means decreasing *x* in NdFeO_3+*x*_). The relatively small variation in the majority carrier density calculated from Mott–Schottky plots, suggests that, in this case, charge compensation occurs through a loss of excess oxygen rather than by an increase in the Fe^4+^ density. The surface segregation of the dopants (as revealed in previous reports^33^ and also in agreement with our XPS analysis) further supports the idea that doping does not induce major modifications in the material bulk (thus implying a limited increase in the bulk Fe^4+^ density).

**Figure 9:**
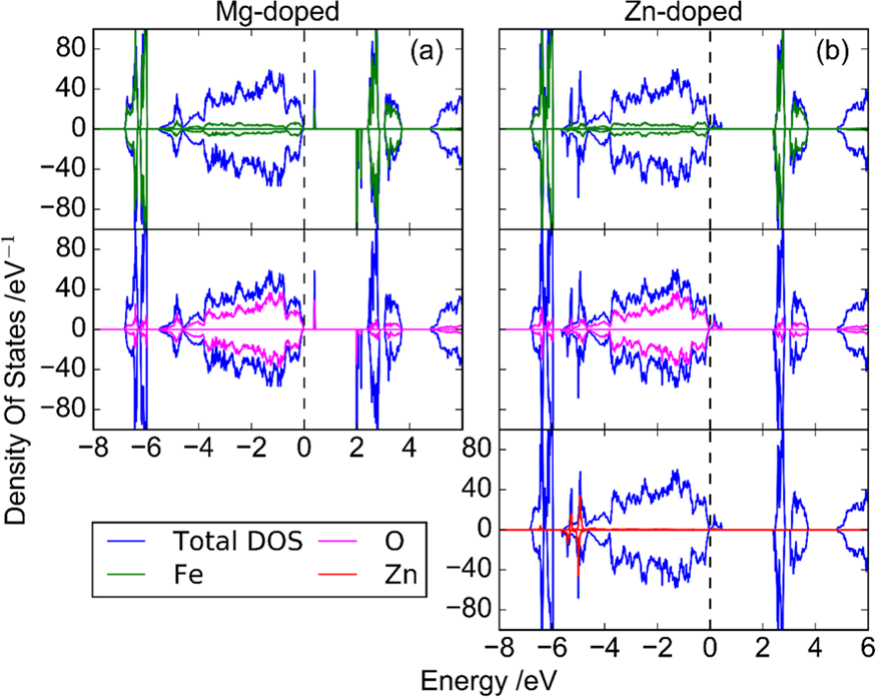
Total and partial density of states for NdFeO_3_ at the PBE + *U* level (*U* (Fe) = 4.0 eV) doped (6.7 at %) with (a) Mg and (b) Zn (the dashed line is the Fermi level, which has been shifted to zero).

DFT calculations can also illustrate the effect of doping on the electronic structure (Figure 9). Doping with Mg (Figure 9a) gives rise to highly localized empty states appearing 0.4 eV above the valence band edge with contributions from both Fe (due to the appearance to Fe^4+^) and O. The generation of Fe^4+^ species is also reflected in the appearance of localized states below the conduction band edge. No significant contributions from Mg states are discernable. In the case of Zn doping (Figure 9b), the new empty states that appear in the band gap just above the valence band edge are hybridized, which means that they are less localized. For both dopants, the appearance of empty states just above the valence band edge explains the enhancement of the p-type semiconducting character of the oxide. Apart from the new states that appear in the band gap, the electronic structure is not severely disturbed with respect to that of pristine NdFeO_3_: the DOS at the band edges is similar in all cases and there is no significant alteration of the calculated band gap (2.4 eV in all cases).

Previous reports have referred to an increase in the mobility of the majority charge carriers with the dopant concentration in certain semiconductors, such as In_2_O_3_^65−67^ and SrTiO_3_.^68^ In the case of perovskites, this increase in mobility has usually been justified by considering the lattice distortion induced by doping. Not in vain, significant variations of the unit cell parameters have been measured for the LaFeO_3_ structure upon incorporation of divalent Zn and Mg ions.^34,50,52,69,70^ The slightly different ionic radii of the dopant cations in comparison with Fe^3+^ in the B position of the host structure and its partial oxidation to Fe^4+^ (with a smaller radius of 58.5 pm) explain this structure distortion. In our case, XRD patterns for the pristine and doped samples also reveal the appearance of some distortion upon doping.

**Figure 10:**
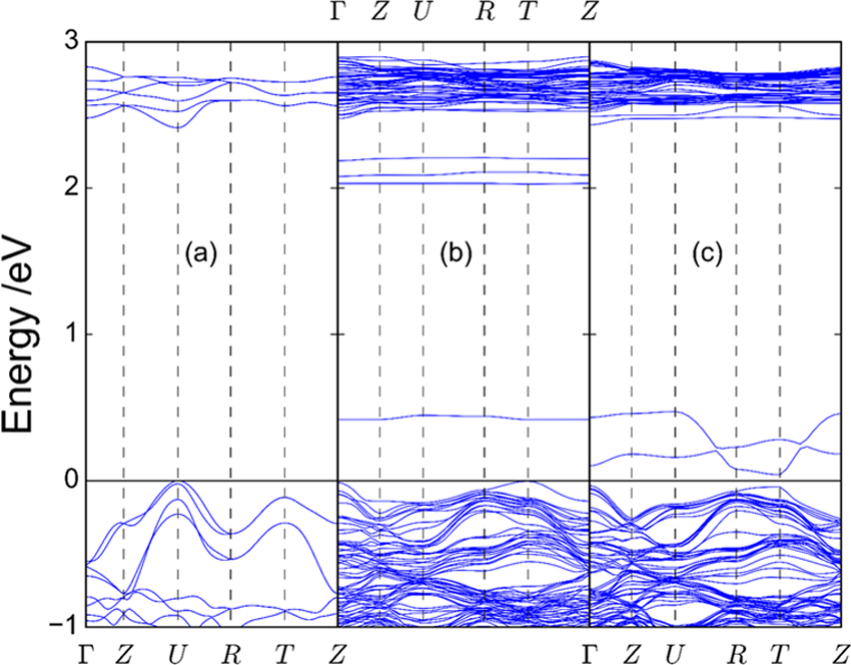
Band structure for (a) NdFeO_3_, (b) Mg-NdFeO_3_, and (c) Zn-NdFeO_3_.

Figure 10 shows the band structure for Mg- and Zn-doped NdFeO_3_, together with that of the pristine material for the sake of comparison. As observed, below the Fermi level the curvature of the bands is not severely altered upon doping. Near the Γ point for doped NdFeO_3_, the curvature of the upper band is comparable to that around the U point for undoped NdFeO_3_. On the other hand, in the conduction band for doped NdFeO_3_, there is a clear flattening of the bands with respect to undoped NdFeO_3_. This implies that the effective mass for the valence band holes is similar in all cases, while the effective mass for conduction band electrons is significantly higher in the case of doped NdFeO_3_ as a consequence of a distortion of the crystal structure. It thus seems that doping with Mg or Zn mainly increases the p-type character of the NdFeO_3_ perovskite through an increase in the hole density. However, there could be another effect. Figures S13 and S14 illustrate the alteration in the charge density induced in neighboring atoms by Mg and Zn dopants, respectively. In both cases, the introduction of Mg or Zn induces an electron density deficiency in a neighboring Fe atom, while only in the case of Mg, it induces an excess of electron density in four of the O atoms surrounding Mg. Previously, it has been concluded that the introduction of Mg or Zn does not cause a significant improvement in the mobility of the majority carriers, lowering that of the minority carriers. As oxygen orbitals mainly contribute to the valence band, the existence of oxygen atoms with an excess of negative charge suggests that the nature of holes for Mg-doped samples could be different from that in Zn-doped samples. This could favor charge separation in the bulk by diminishing recombination. This factor does not operate in the case of Zn-doped samples, which explains their slightly lower performance.

## 4 Conclusions

In summary, we have shown that NdFeO_3_ thin-film electrodes prepared by a layer-by-layer coating of conducting glass substrates by a citrate-route sol–gel method, followed by heat treatment, behave as photocathodes for oxygen and water reduction in alkaline media. Their photoactivity, especially for the oxygen reduction reaction, can be significantly enhanced by doping with either Mg^2+^ or Zn^2+^. In fact, for the optimum doping level (5 at % for both Mg^2+^ and Zn^2+^), the photocurrent due to the oxygen reduction reaction multiplies by factors of 3.4 and 2.8, respectively. This important enhancement of the *p*-type behavior (further confirmed by DFT calculations) of NdFeO_3_ has been attributed to an increase in the majority carrier density (as demonstrated by Mott–Schottky measurements). This results from a dopant-induced increase in the density of VB holes in the NdFeO_3_ lattice caused by charge compensation when Fe^3+^ centers are substituted by divalent Mg^2+^ and Zn^2+^ cations. On the other hand, doping has not been observed to affect in a significant way the mobility of the majority carrier.

It has been demonstrated that NdFeO_3_ presents a photoelectrochemical performance similar to that of the widely studied LaFeO_3_ perovskite. This turns NdFeO_3_ into an attractive candidate material for photocathodes in tandem devices due to its environmental friendliness and the relatively high earth abundance of its constituent elements (Nd is as abundant as La in the Earth’s crust). As in the case of LaFeO_3_, NdFeO_3_ also presents a very positive potential onset for cathodic photocurrents and a competitive narrow band-gap value. Doping with Mg and Zn leads to an improvement of the photogenerated electron transfer kinetics to promote hydrogen evolution reaction from water, although the responses are always modest. Further enhancement is thus needed and, in this respect, a promising combination of an extraction layer with a proper metal co-catalyst has been tested and optimized in our laboratory and it will be reported elsewhere.

Finally, DFT calculations of the electronic structure of NdFeO_3_ at the PBE + *U* level have provided a band-gap value close to that found experimentally and they have confirmed the p-semiconducting character of this material. In addition, the calculated band structure shows a rather flat conduction band, indicating low electron mobility. Importantly, the effective masses of carriers calculated from the band structures indicate a significant anisotropy in mobility, both for electrons and holes, pointing to the importance of nanostructuring. DFT calculations have also shed light on the effects of doping on the electronic structure. The importance of theoretical calculations in guiding the search for effective enhancement strategies in photoelectrochemical material is thus illustrated.

## Supporting Information Available

XRD, SEM, XPS, and UV–vis analysis experimental details; optimization of the NdFeO_3_ thin-film electrode thickness; additional FESEM characterization; additional XPS characterization; optical characterization; additional (photo)electrochemical characterization; and additional DFT results (PDF)
