# Supplementary material for: Photoelectrochemical Behavior and Computational Insights for Pristine and Doped NdFeO3 Thin-Film Photocathodes
Source: ACS Appl Mater Interfaces. 2021 Mar 17;13(12):14150–9. doi: 10.1021/acsami.0c21792 (PMC8485327; doi:10.1021/acsami.0c21792)
Supplement: Supplementary file 1 — am0c21792_si_001.pdf [file am0c21792_si_001.pdf]

Supporting Information for:

Photoelectrochemical Behavior and Computational Insights for Pristine and Doped $NdFeO_3$ Thin Film Photocathodes

*Javier Quiñonero,[a] Francisco J. Pastor,[a] José M. Orts,[a,b] Roberto Gómez[a,b]**

[a]Departament de Química Física i [b]Institut Universitari d'Electroquímica

Universitat d'Alacant, Apartat 99, E-03080 Alicante, Spain

**Corresponding author. Tel: +34 96 590 3748; email address: roberto.gomez@ua.es*

Page

1. XRD, SEM, XPS and UV-vis analysis experimental details ……………… S2

2. Optimization of the $NdFeO_3$ thin film electrode thickness……………..… S3

3. Additional FESEM characterization…………………………………....… S5

4. Additional XPS characterization………………………......………...…. S7

5. Optical characterization……………………………………...……… S9

6. Additional (photo)electrochemical characterization……………...………. S10

7. Additional DFT results………………...…………………………………. S14

1. XRD, SEM, XPS and UV-vis analysis experimental details

The crystal structure of the $NdFeO_3$ deposits was determined by XRD, using a Bruker, D8-Advance X-ray diffractometer operating at room temperature with Cu-Kα radiation (λ = 1.5416 Å) at 40 kV and 40 mA. The angular velocity was 0.5º·min^{-1} within a 2θ range between 20º and 70º.

A SEM study was carried out to characterize the surface morphology of the films using a ZEISS Merlin VP Compact field emission scanning electron microscope (FESEM).

For the film surface composition study, XPS experiments were done with a Thermo-Scientific K-Alpha XPS spectrometer equipped with a monochromatic Al-Kα source (1486.6 eV), operating at 15 kV and 10 mA.

The optical properties of the films were studied by solid-state UV-vis spectroscopy, using a Shimadzu UV-2401 PC spectrophotometer equipped with an integrating sphere and working in the absorbance mode.

2. Optimization of the $NdFeO_3$ thin film electrode thickness

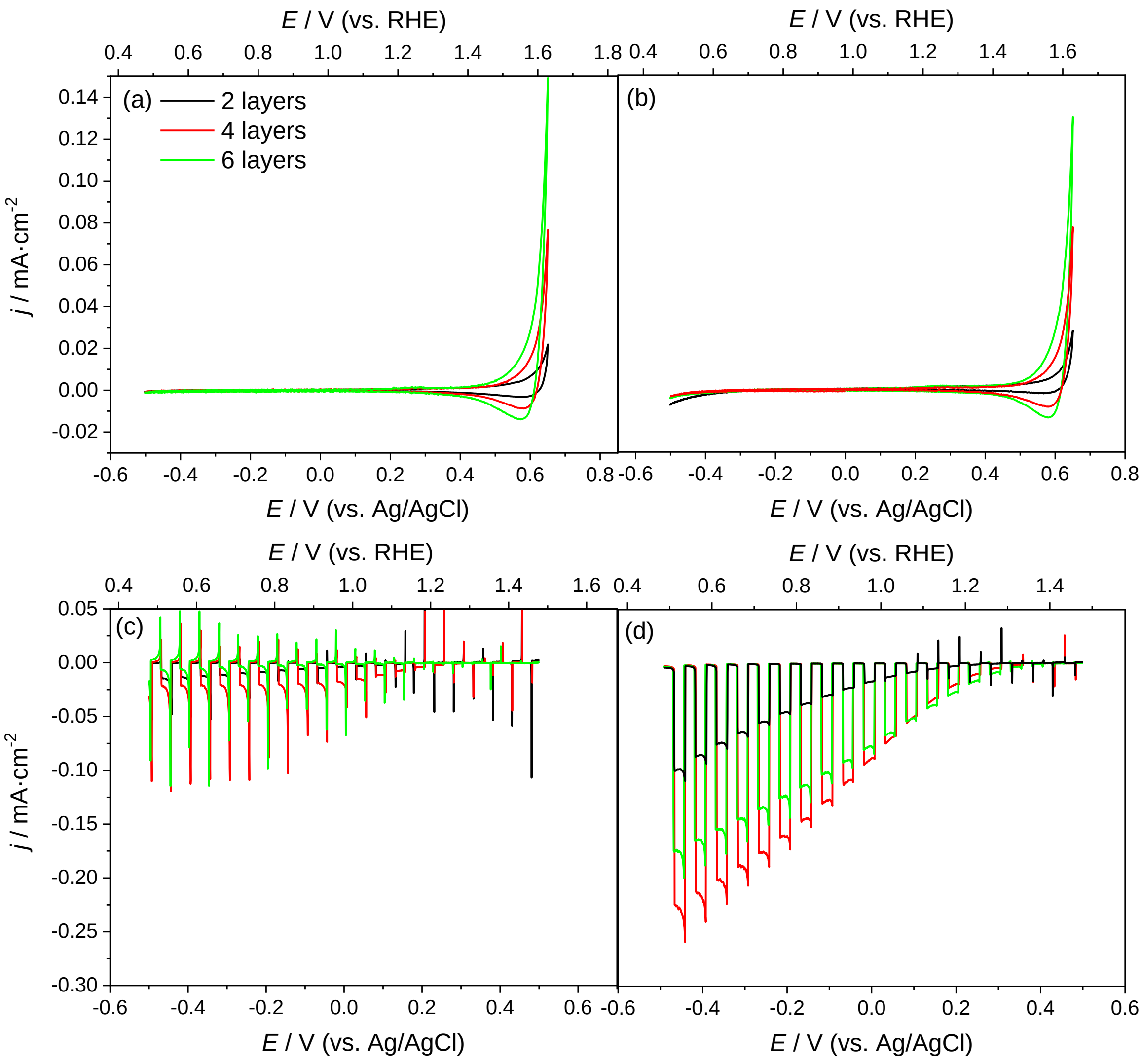

Figure S1. Cyclic voltammograms in the dark in (a) N_2- and (b) O_2-purged 0.1 M NaOH for 2–, 4– and 6–layer pristine $NdFeO_3$ electrodes (scan rate: 20 $mV{\cdot}s^{-1}$). Linear scan voltammograms under transient illumination in (c) N_2- and (d) O_2-purged 0.1 M NaOH for 2–, 4– and 6–layer pristine $NdFeO_3$ electrodes (scan rate: 5 $mV{\cdot}s^{-1}$).

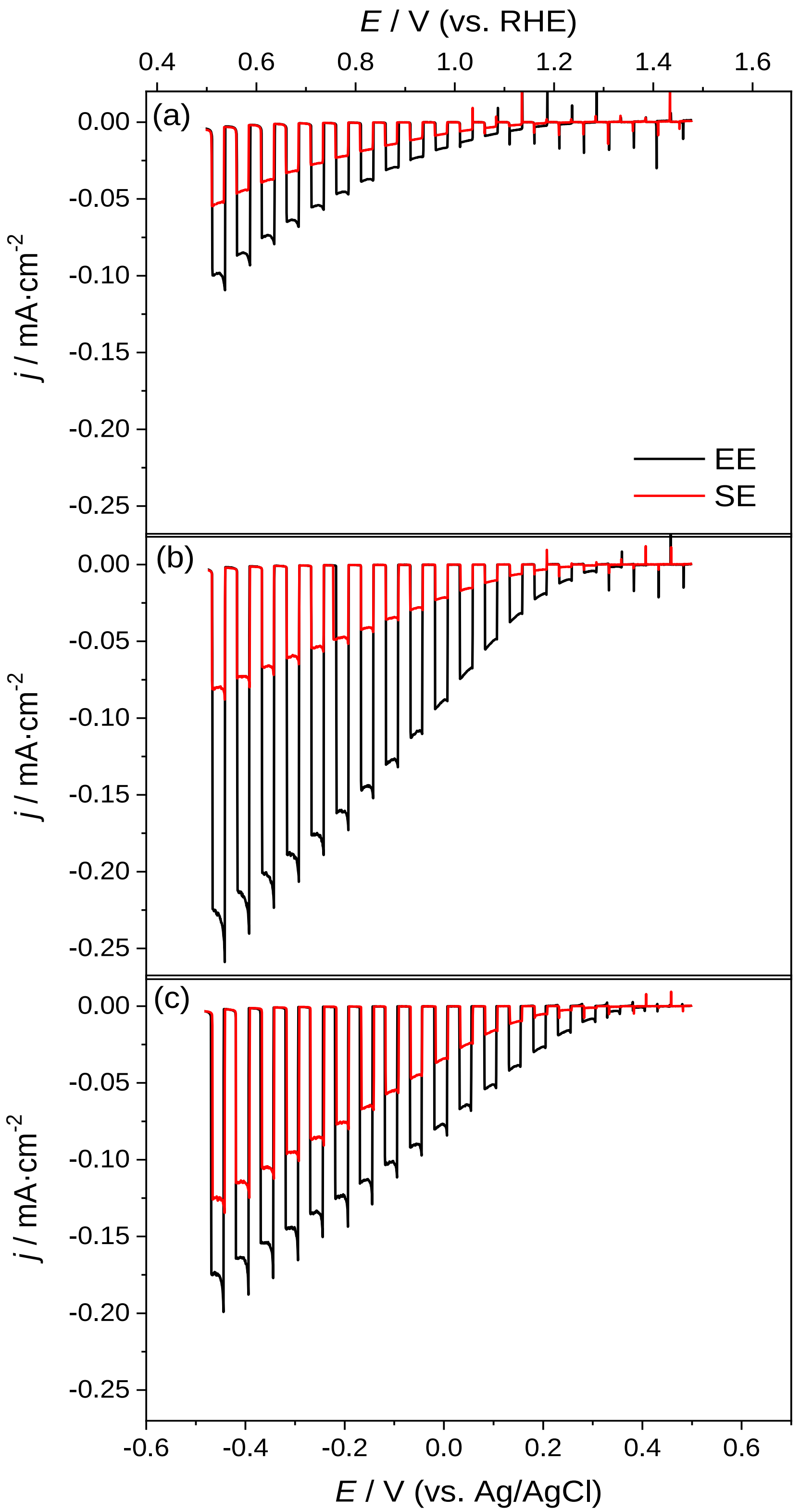

Figure S2. Linear scan voltammograms for (a) 2–, (b) 4– and (c) 6–layer pristine $NdFeO_3$ electrodes under transient electrolyte-electrode (EE) and substrate-electrode (SE) illumination (scan rate: 5 $mV \cdot s^{-1}$), in O_2-purged 0.1 M NaOH.

3. Additional FESEM characterization

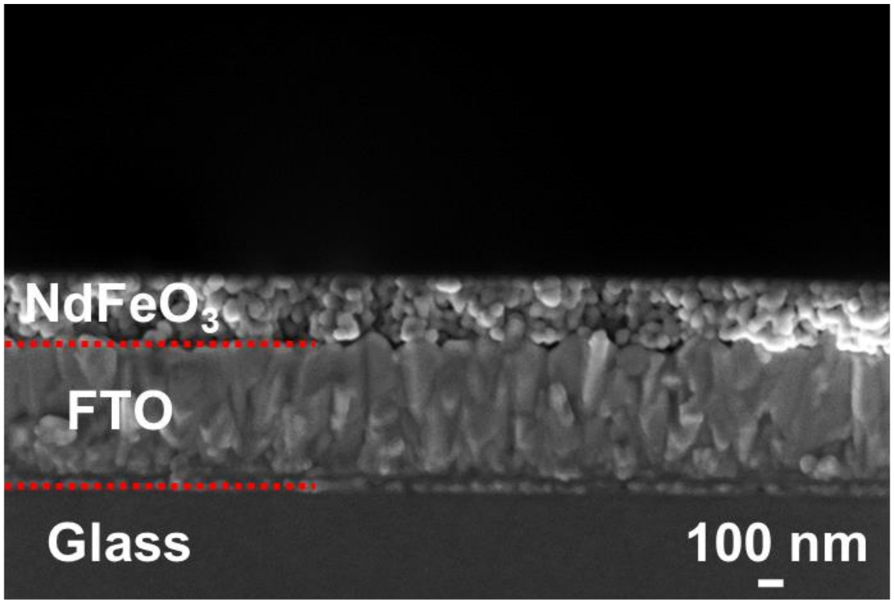

Figure S3. Cross-sectional FESEM image for an FTO/$NdFeO_3$ electrode.

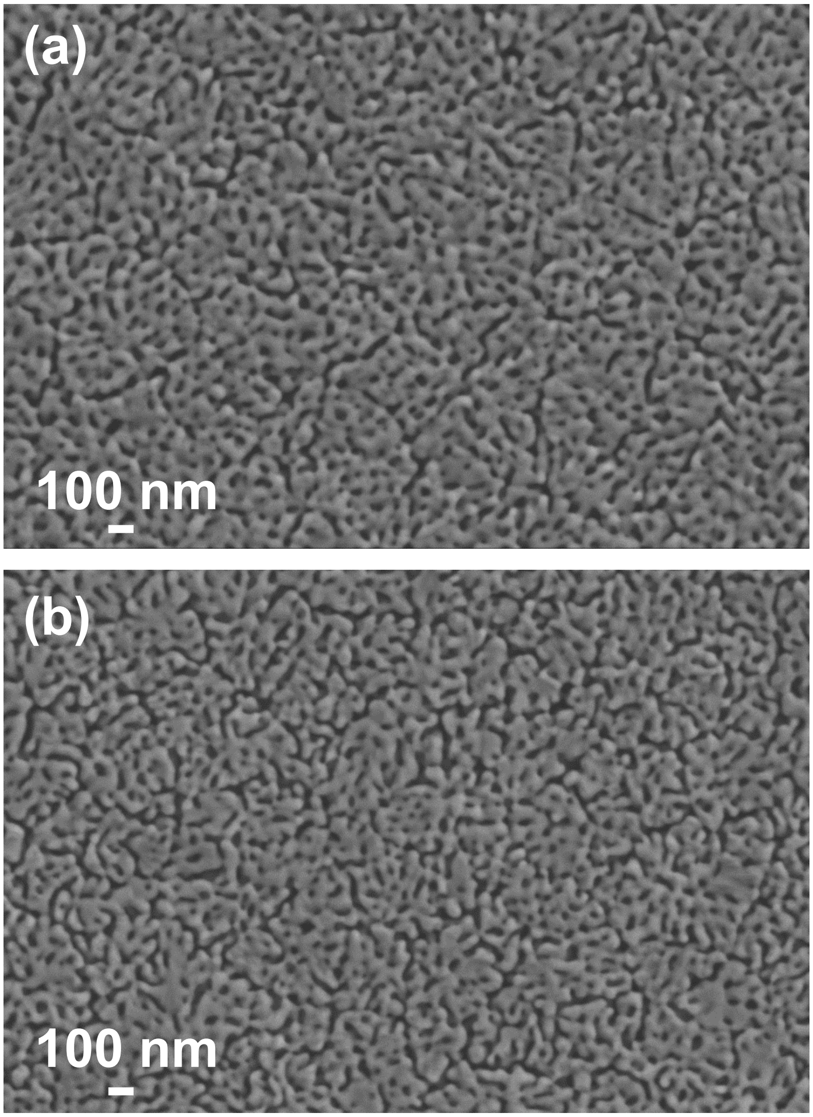

Figure S4. FESEM image corresponding to the top view of (a) Mg (5 at%)-$NdFeO_3$ and (b) Zn (5 at%)-$NdFeO_3$ films on FTO.

4. XPS characterization

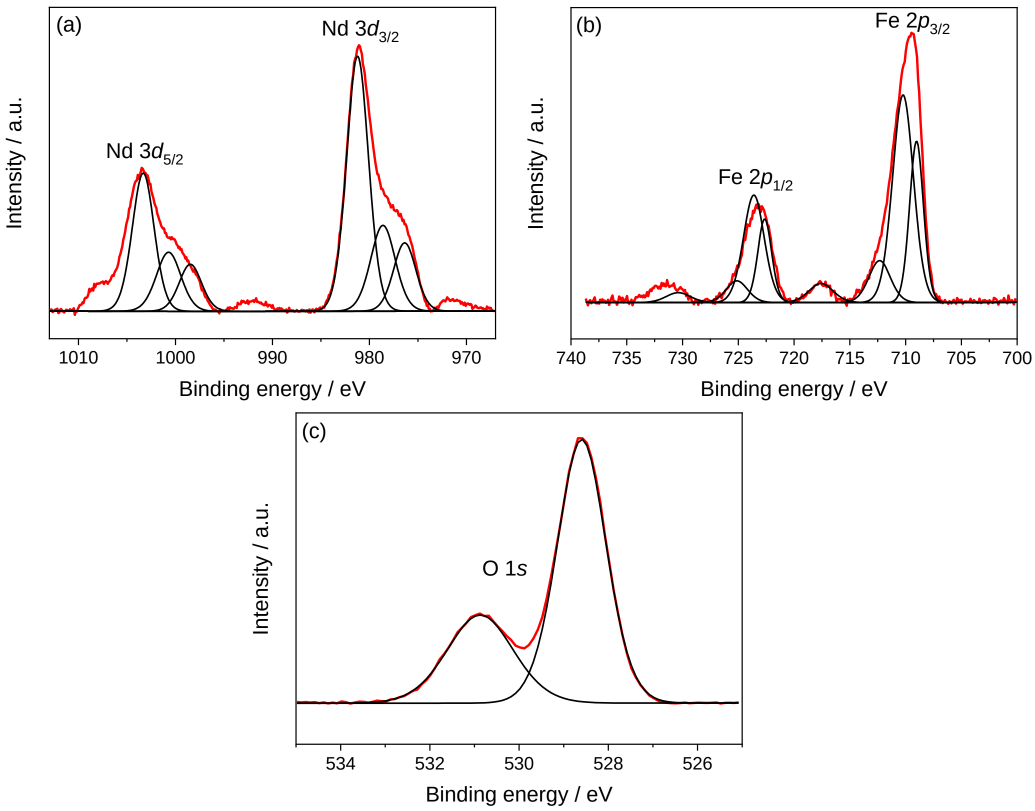

Figure S5. (a) Nd 3*d*, (b) Fe 2*p* and (c) O 1*s* XPS spectra (red line), and their corresponding deconvolutions (black lines), for a Mg (5 at%)-$NdFeO_3$ film.

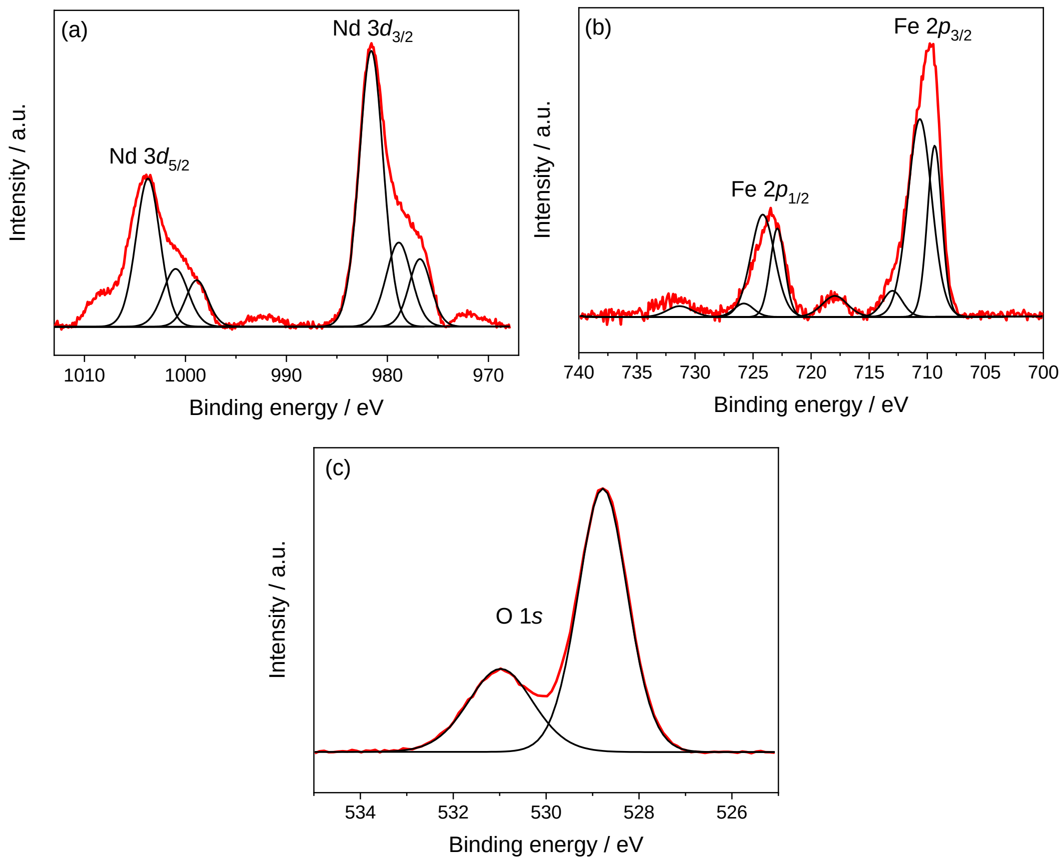

Figure S6. (a) Nd 3*d*, (b) Fe 2*p* and (c) O 1*s* XPS spectra (red line), and their corresponding deconvolutions (black lines), for a Zn (5 at%)-$NdFeO_3$ film.

Table S1. Atomic ratios as obtained by XPS analysis for $NdFeO_3$, Mg (5 at%)-$NdFeO_3$ and Zn (5 at%)-$NdFeO_3$. In parentheses, the corresponding theoretical values in the bulk.

	**ATOMIC RATIO (obtained by XPS analysis)**			
	**Nd / Fe**	**Mg / Fe**	**Zn / Fe**	**OH / O_{latt}**
**$NdFeO_3$**	1.5 (1.0)	-	-	0.37
**Mg (5 at%)-$NdFeO_3$**	1.4 (1.05)	0.27 (0.05)	-	0.45
**Zn (5 at%)-$NdFeO_3$**	1.4 (1.05)	-	0.32 (0.05)	0.41

5. Optical characterization

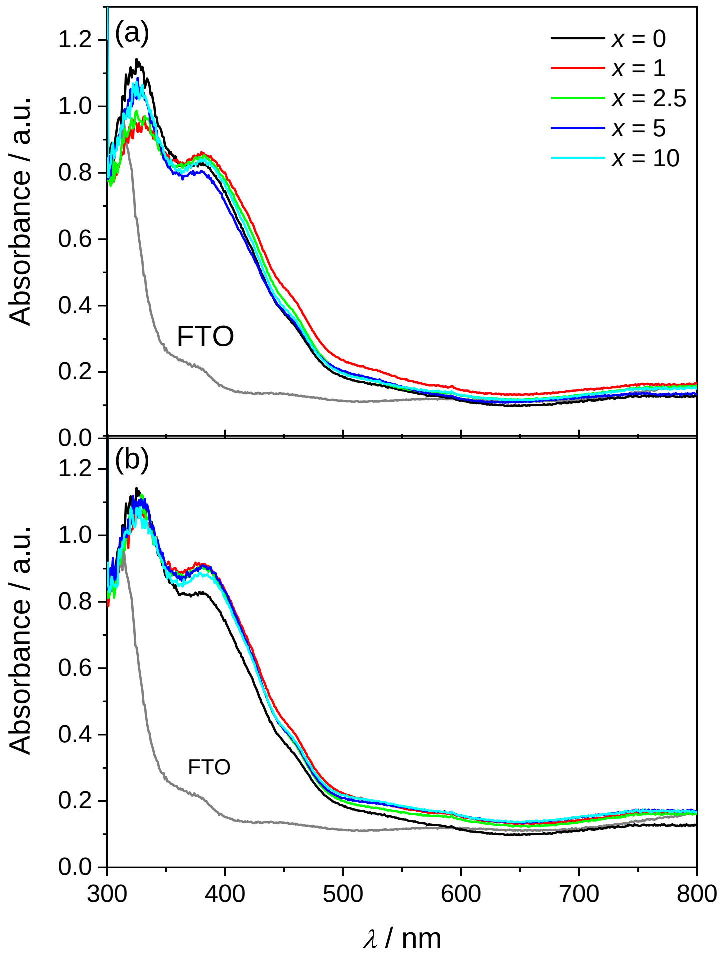

Figure S7. UV-visible absorbance spectra for (a) Mg (*x* at%)-$NdFeO_3$ and (b) Zn (*x* at%)-$NdFeO_3$ films.

6. Additional (photo)electrochemical characterization

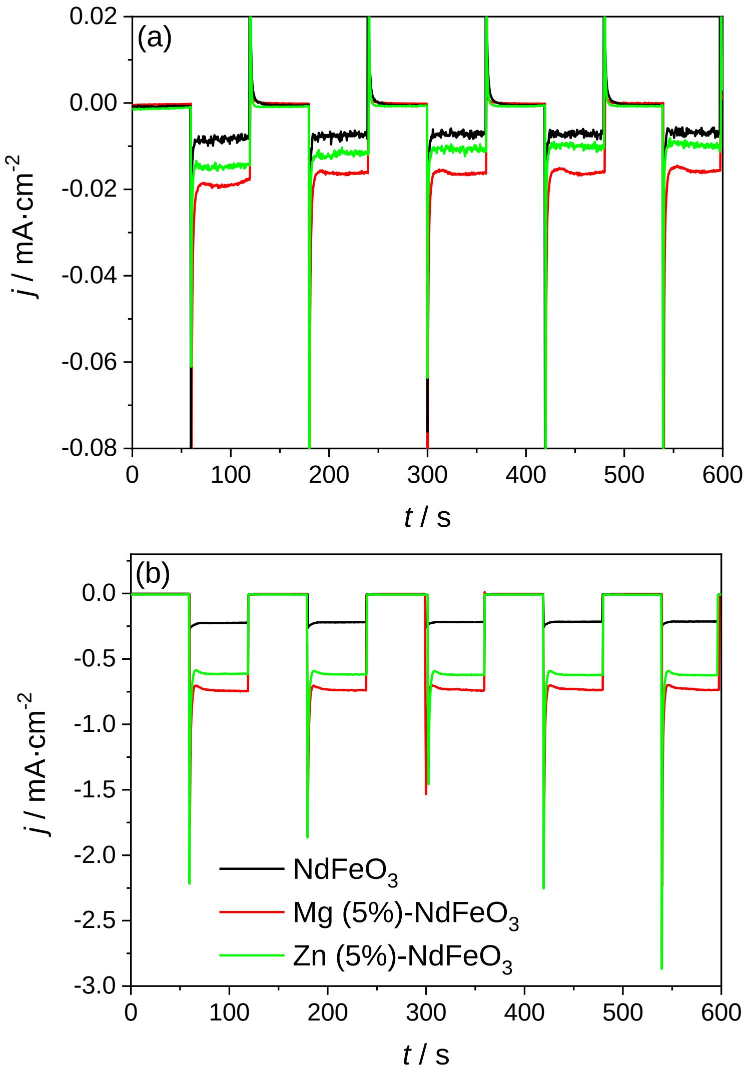

Figure S8. Stationary photocurrent at 0.1 V for pristine, Mg (5 at%)- and Zn (5 at%)-doped $NdFeO_3$ electrodes in (a) N_2- and (b) O_2-purged 0.1 M NaOH electrolyte.

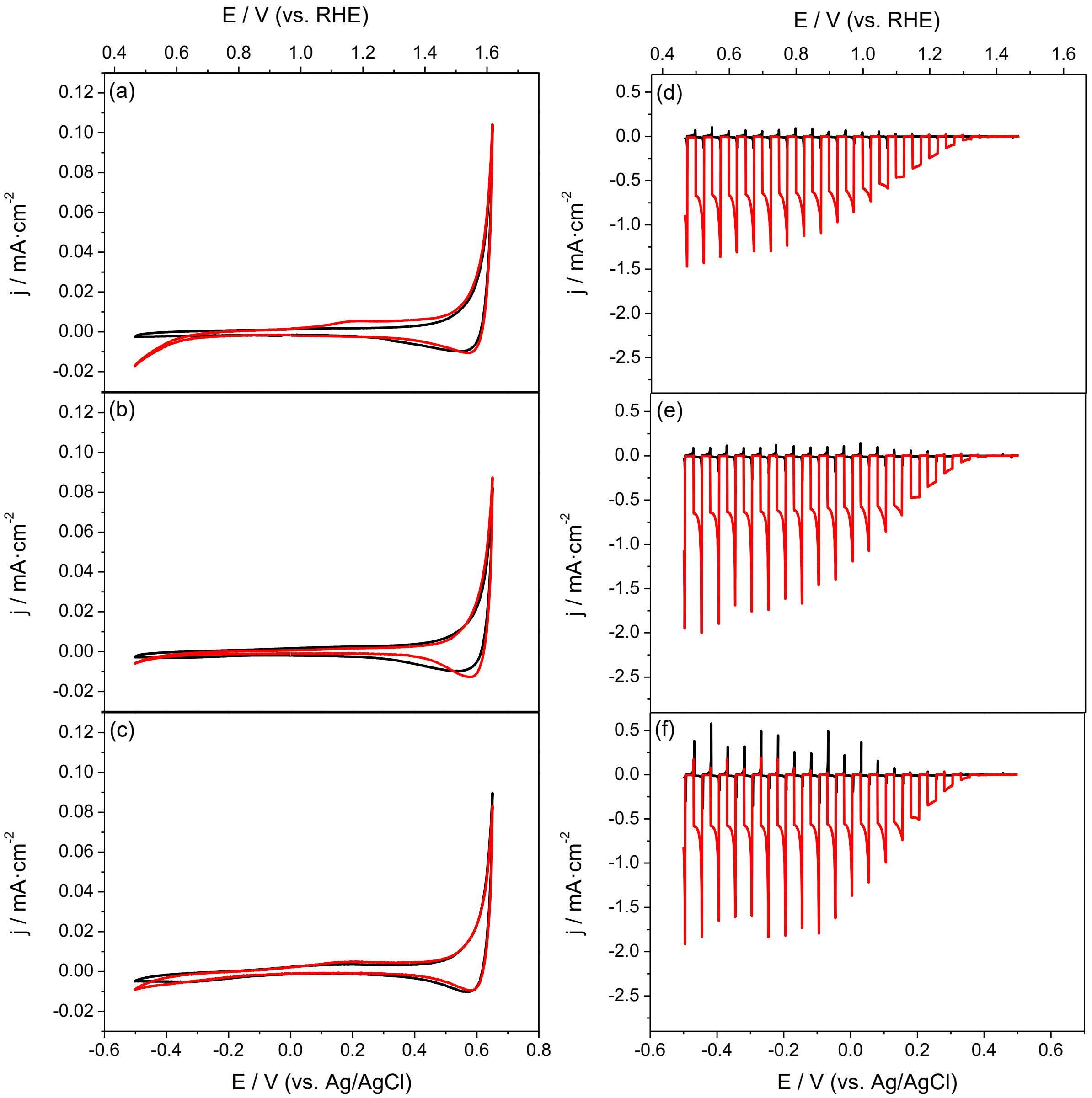

Figure S9. Cyclic voltammograms in the dark in N_2- (black line) and O_2-purged (red line) 0.1 M NaOH for (a) Mg (1 at%)-$NdFeO_3$, (b) Mg (2.5 at%)-$NdFeO_3$ and (c) Mg (10 at%)-$NdFeO_3$ electrodes (scan rate: 20 $mV{\cdot}s^{-1}$). Linear scan voltammograms under transient illumination in N_2- (black line) and O_2-purged (red line) 0.1 M NaOH for (d) Mg (1 at%)-$NdFeO_3$, (e) Mg (2.5 at%)-$NdFeO_3$ and (f) Mg (10 at%)-$NdFeO_3$ electrodes (scan rate: 5 $mV{\cdot}s^{-1}$).

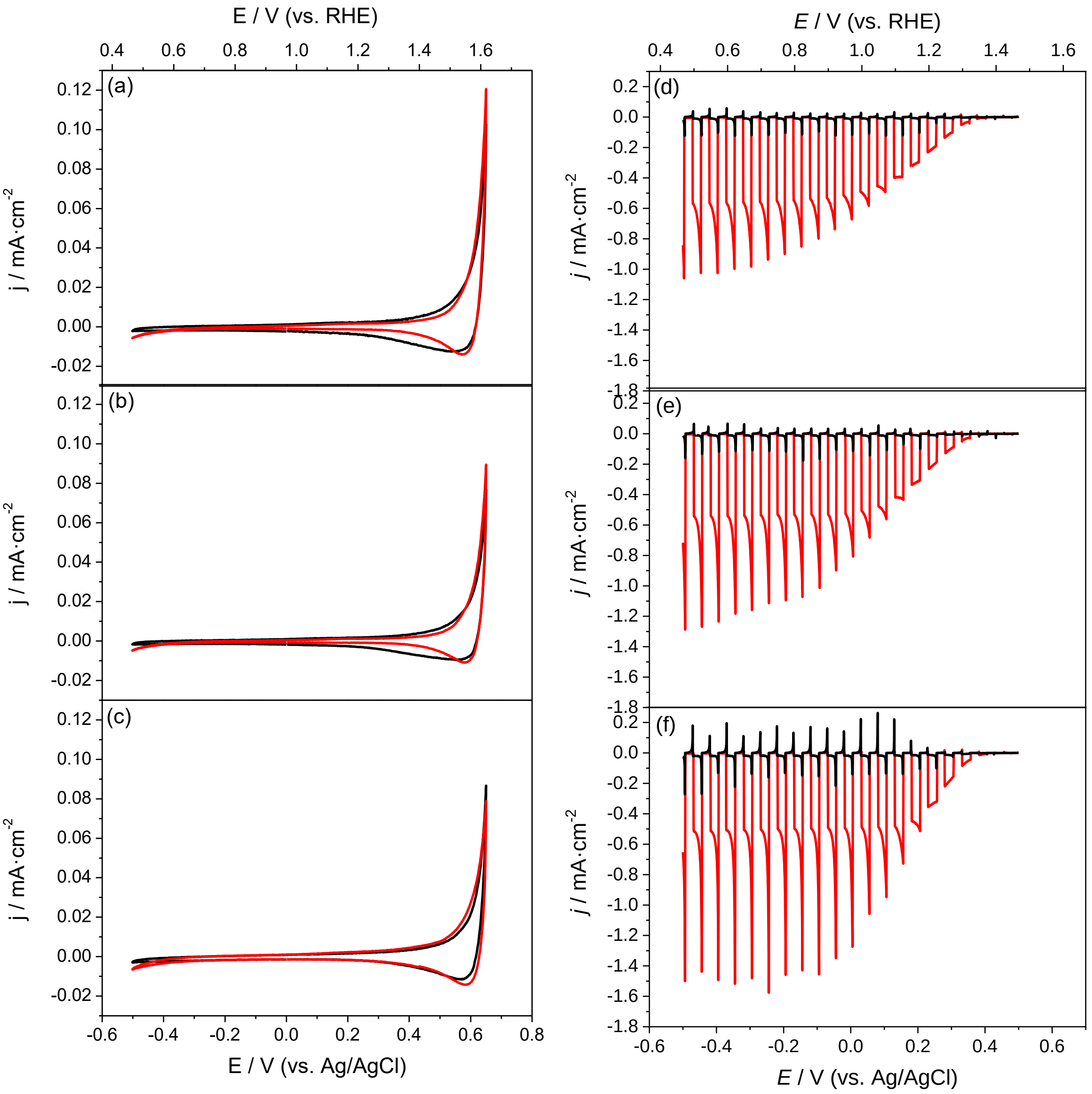

Figure S10. Cyclic voltammograms in the dark in N_2- (black line) and O_2-purged (red line) 0.1 M NaOH for (a) Zn (1 at%)-$NdFeO_3$, (b) Zn (2.5 at%)-$NdFeO_3$ and (c) Zn (10 at%)-$NdFeO_3$ electrodes (scan rate: 20 $mV\cdot s^{-1}$). Linear scan voltammograms under transient illumination in N_2- (black line) and O_2-purged (red line) 0.1 M NaOH for (d) Zn (1 at%)-$NdFeO_3$, (e) Zn (2.5 at%)-$NdFeO_3$ and (f) Zn (10 at%)-$NdFeO_3$ electrodes (scan rate: 5 $mV\cdot s^{-1}$).

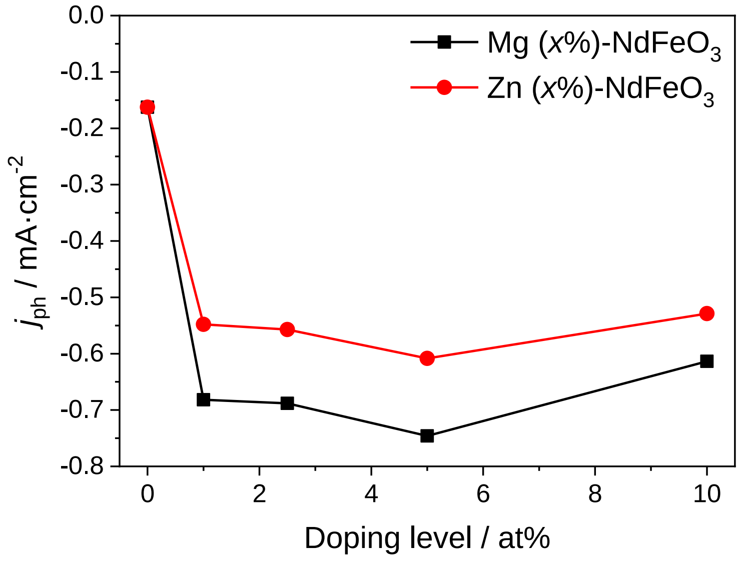

Figure S11. Stationary photocurrent at 0.1 V as a function of doping level (*x*) for Mg (*x* at%)- and Zn (*x* at%)-doped $NdFeO_3$ electrodes.

Table S2. Absolute values of the slopes of the linear region in the Mott-Schottky plots shown in Fig. 5 for pristine and doped $NdFeO_3$ electrodes, in the dark and under illumination.

	$\lvert 10^{-10}\cdot$slope$\rvert$ / $F^{-2}\cdot cm^{-4}\cdot V^{-1}$	
	**Dark**	**Illumination**
**$NdFeO_3$**	30.0	25.9
**Mg (1 at%)-$NdFeO_3$**	29.3	22.1
**Mg (2.5 at%)-$NdFeO_3$**	24.4	14.9
**Mg (5 at%)-$NdFeO_3$**	6.1	4.0
**Mg (10 at%)-$NdFeO_3$**	5.3	3.8
**Zn (1 at%)-$NdFeO_3$**	28.2	14.8
**Zn (2.5 at%)-$NdFeO_3$**	17.5	13.2
**Zn (5 at%)-$NdFeO_3$**	9.7	4.7
**Zn (10 at%)-$NdFeO_3$**	5.1	2.7

7. Additional DFT results

For the HSE functional, the values of the mixing parameter for exact Fock exchange and the screening parameter were 0.15 and 0.2 $Å^{-1}$, respectively. A single-point calculation has been made to obtain the DOS (for the geometry optimized at the PBE+U level) with an energy cut-off of 400 eV, a 5x5x3 k-mesh and an electronic convergence criterion of 10^{-5} eV.

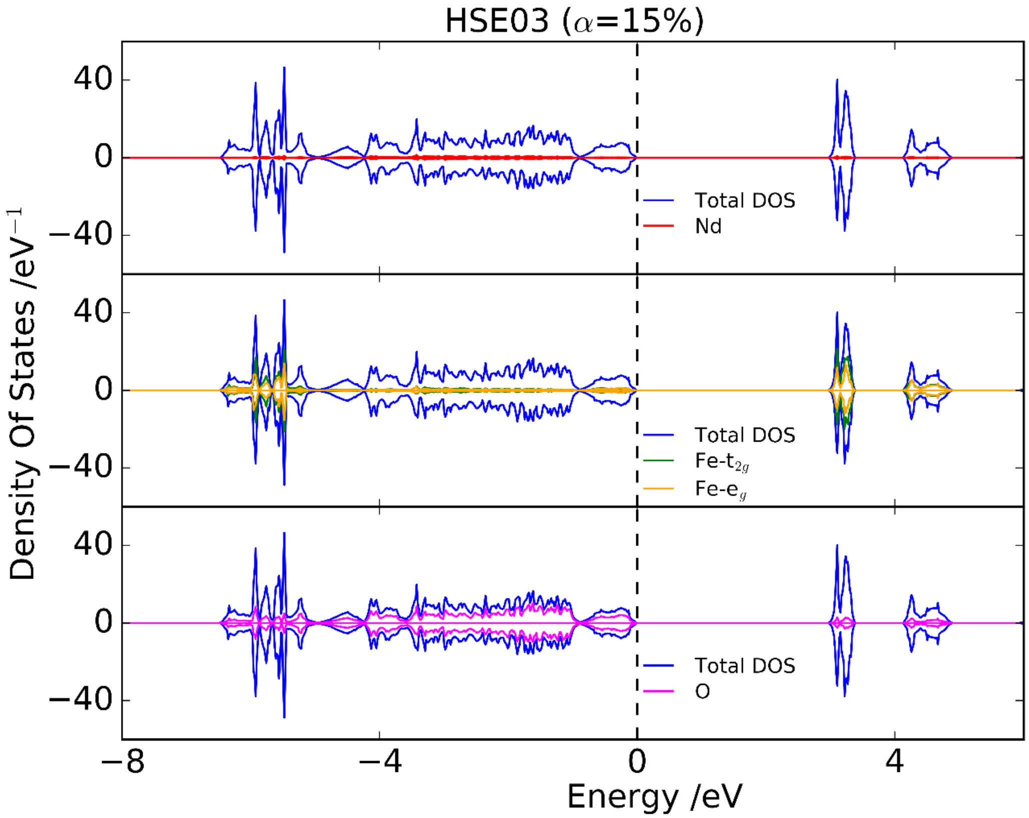

Figure S12. Total and partial density of states for $NdFeO_3$ at HSE03 (α = 15%) (the dashed line is the Fermi level, which has been shifted to zero).

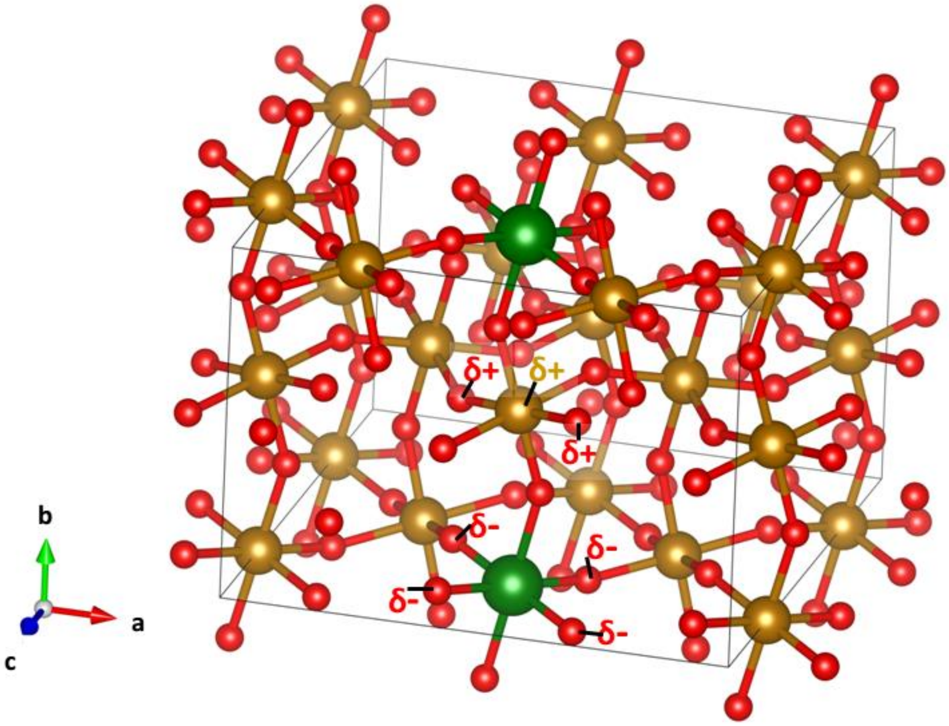

Figure S13. Charge distribution around de Mg atom based on Bader charge analysis. Nd atoms and bond with Nd have been removed for simplicity. Mg: green; O: red; Fe: gold.

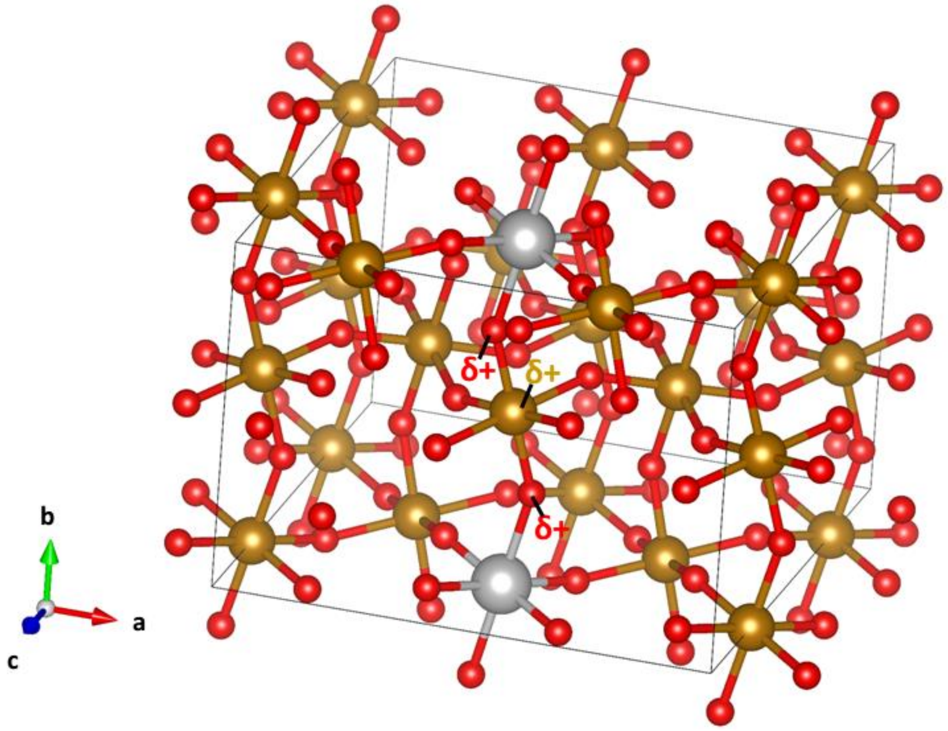

Figure S14. Charge distribution around de Zn atom based on Bader charge analysis. Nd atoms and bond with Nd have been removed for simplicity. Zn: grey; O: red; Fe: gold.

Table S3. Calculated band gap and effective masses of carriers in $NdFeO_3$ from band structure through HSE03 calculations.

Calculation	Band gap / eV	Charge carrier	m^*_a / m_e	m^*_b / m_e	m^*_c / m_e
**HSE03**	3.0	Electron	10.0	2.9	11.4
		Hole	1.4	1.5	10.1
